# Effects of Maternal Zinc Deficiency and Post-Weaning Zinc Status on Neuroendocrine Markers Associated with Pubertal Regulation in Female Rat Offspring

**DOI:** 10.3390/ijms27167479

**Published:** 2026-08-21

**Authors:** Meltem Gumus, Buse Gunaydın-Turker, Nilufer Akgun-Unal, Elif Gulbahce-Mutlu, Saltuk Bugra Baltaci, Omer Unal, Rasim Mogulkoc, Abdulkerim Kasim Baltaci

**Affiliations:** 1 Medical Faculty, Department of Child Health and Diseases, Selcuk University, 42250 Konya, Türkiye; meltemdorum@gmail.com; 2 Medical Faculty, Department of Physiology, Erciyes University, 38030 Kayseri, Türkiye; busegunaydin@erciyes.edu.tr; 3 Medical Faculty, Department of Biophysics, Ondokuz Mayis University, 55139 Samsun, Türkiye; nilufer.akgununal@omu.edu.tr; 4 Medical Faculty, Department of Medical Biology, KTO Karatay University, 42020 Konya, Türkiye; elifgulbahcemutlu@gmail.com; 5 Medical Faculty, Department of Physiology, Istanbul Medipol University, 34810 Istanbul, Türkiye; bugrabaltaci@icloud.com; 6 Medical Faculty, Department of Physiology, Samsun University, 55420 Samsun, Türkiye; omer.unal@samsun.edu.tr; 7 Medical Faculty, Department of Physiology, Selcuk University, 42250 Konya, Türkiye; mogulkoc@selcuk.edu.tr

**Keywords:** maternal zinc deficiency, nutrition, puberty, hypothalamus, hormonal parameters, rat

## Abstract

This study investigated the effects of maternal zinc deficiency and post-weaning zinc status on neuroendocrine markers associated with pubertal regulation in female rat offspring. A total of 40 female rats were divided into four groups: zinc deficiency (G1; MZD + ZD), standard diet (G2; MZD + SD), zinc supplementation (G3; MZD + ZnS), and control (G4; CON). Groups G1–G3 were obtained from mothers with zinc deficiency, while G4 was obtained from mothers fed a standard diet. The procedures lasted 45 days. GATAD1, kisspeptin, GnRH, and NPY gene expressions in hypothalamic tissues were analyzed by RT-PCR; serum kisspeptin, GnRH, FSH, LH, leptin, and NPY levels were analyzed by ELISA. The lowest GATAD1, kisspeptin, and NPY gene expressions were found in groups G1 and G2 (*p* < 0.05), while the lowest GnRH expression was found in group G1 (*p* < 0.05). Serum zinc, kisspeptin, GnRH, LH, and leptin levels were lowest in G1 (*p* < 0.05) and highest in G3 and G4 (*p* < 0.05). Similarly, FSH and NPY levels were low in G1 and G2 (*p* < 0.05), and high in G3 and G4 (*p* < 0.05). The findings suggest that maternal zinc deficiency negatively impacts puberty-related neuroendocrine markers, and that zinc supplementation may correct these markers.

## 1. Introduction

Zinc is an essential micronutrient involved in critical biological processes such as growth, cell division, and endocrine regulation, constituting over 3000 proteins [1,2]. Maternal zinc deficiency during fetal development has been associated with fetal growth retardation, losses, and congenital malformations [3,4]. Inadequate zinc intake during the pre-pubertal period is known to increase protein oxidation, suppress gonadotropin secretion, and suppress LH levels [5].

Kisspeptin, an important regulator of reproductive function, activates GnRH neurons in humans and animals, enabling the onset of puberty [6,7]. GATAD1 is also one of the factors that plays a role in the neuroendocrine regulation of pubertal development through epigenetic mechanisms affecting genes located in the hypothalamus-pituitary-gonadal axis [8]. Therefore, there is a direct link between zinc finger proteins and the reproductive axis. The reproductive system and appetite control mechanisms are managed by common neuronal networks at the hypothalamic level [9,10]. Since the organism’s energy status directly affects the maturation of the reproductive axis, there is a critical relationship between nutrition and puberty [10]. Leptin is a key metabolic signal linking energy availability with reproductive function. Current evidence suggests that leptin is required for normal pubertal maturation; however, it is generally considered a permissive factor that enables activation of the reproductive axis rather than a sole initiator of puberty [11,12]. Similarly, Neuropeptide Y (NPY), a potent appetite stimulant, integrates metabolic and reproductive signals in the neural network [13,14]. The interaction between leptin, NPY, and kisspeptin neurons forms the basis of this neuroendocrine modulation [15]. Zinc deficiency leads to symptoms similar to Anorexia Nervosa, such as loss of appetite, weight loss, and growth retardation [13]. In this process, zinc is active in the regulation of appetite-regulating peptides NPY and galanin [13]. Furthermore, zinc has been shown to have a direct effect on leptin secretion from adipose tissue and Ob mRNA content [16,17,18].

Zinc may play a significant role in the regulation of leptin, NPY, GnRH, and kisspeptin based on the present findings. The aim of this study was to investigate the potential effects of zinc on molecular pathways between neuroendocrine markers involved in the onset of puberty and dietary regulation.

## 2. Results

### 2.1. Serum Zinc Levels

**Figure 1:**
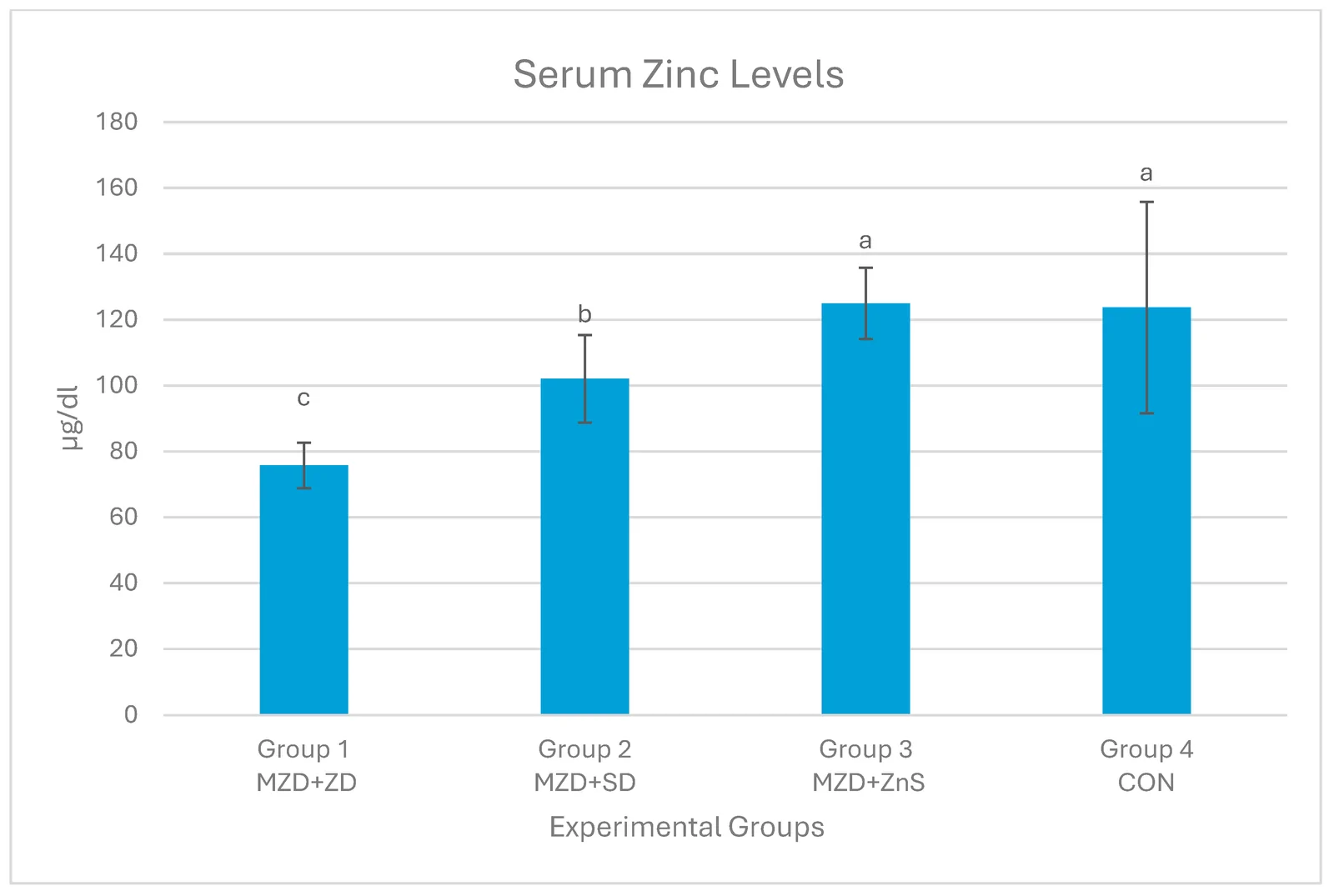
Serum zinc levels of the study groups. Different lowercase letters above the data points indicate statistically significant differences among groups (*p* < 0.05). Groups marked with the same letter are not significantly different, whereas groups marked with different letters show significant differences. Specifically, in this study, the letter “a” indicates the group with the highest expression level (significantly higher than the groups marked with “b”), while the letter “b” indicates the group with a lower expression level (significantly lower than the groups marked with “a”).

The lowest serum zinc levels in our study were observed in G1 (*p* < 0.05), and the highest serum zinc levels were observed in G3 and G4 (*p* < 0.05). Serum zinc levels in G2 were higher than in G1 (*p* < 0.05) and significantly lower than in G3 and G4 (*p* < 0.05, Figure 1 and Table 1).

### 2.2. Hypothalamic RT-PCR Findings

**Figure 2:**
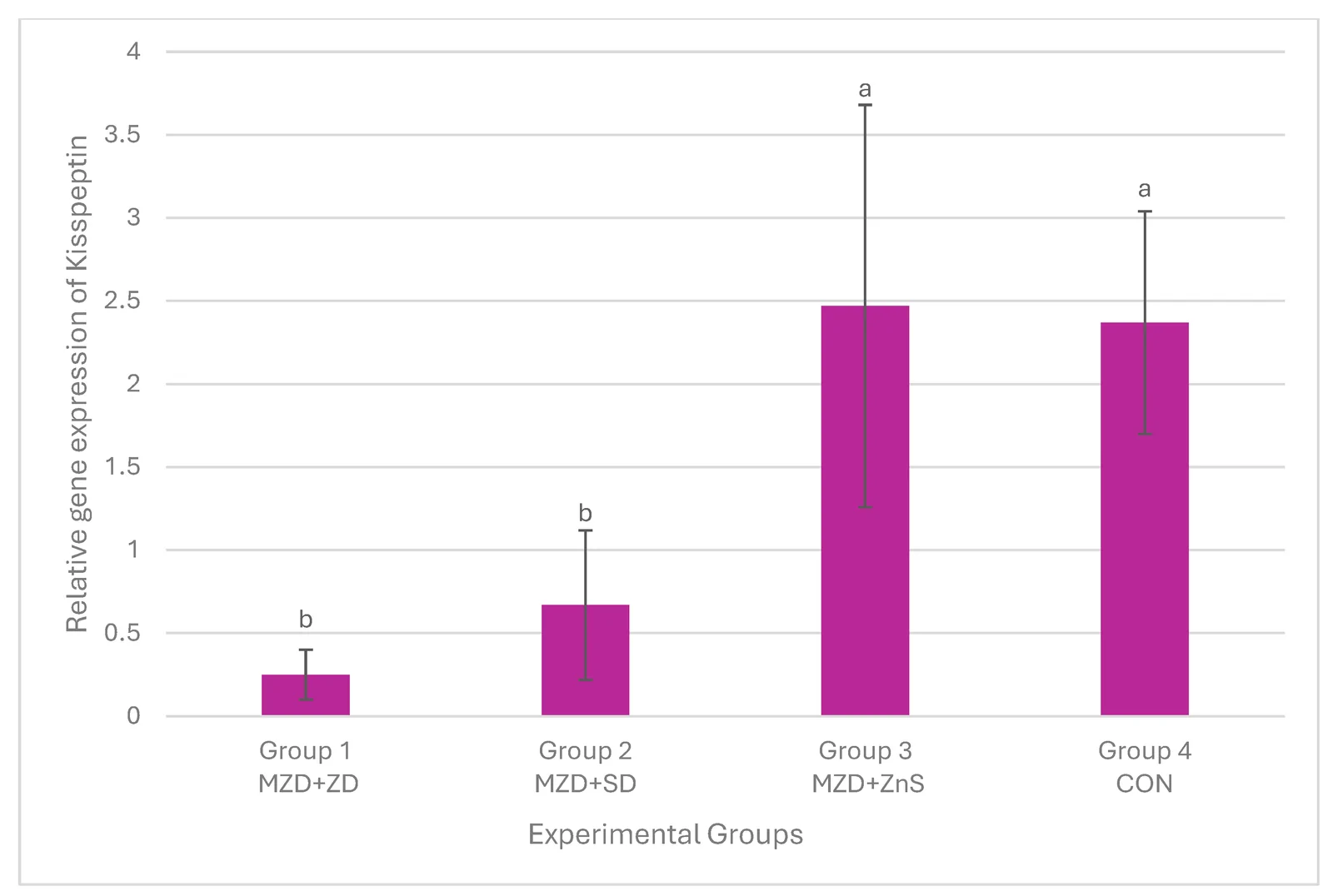
Hypothalamic kisspeptin gene expression levels of study groups. Different lowercase letters above the data points indicate statistically significant differences among groups (*p* < 0.05). Groups marked with the same letter are not significantly different, whereas groups marked with different letters show significant differences. Specifically, in this study, the letter “a” indicates the group with the highest expression level (significantly higher than the groups marked with “b”), while the letter “b” indicates the group with a lower expression level (significantly lower than the groups marked with “a”).

**Figure 3:**
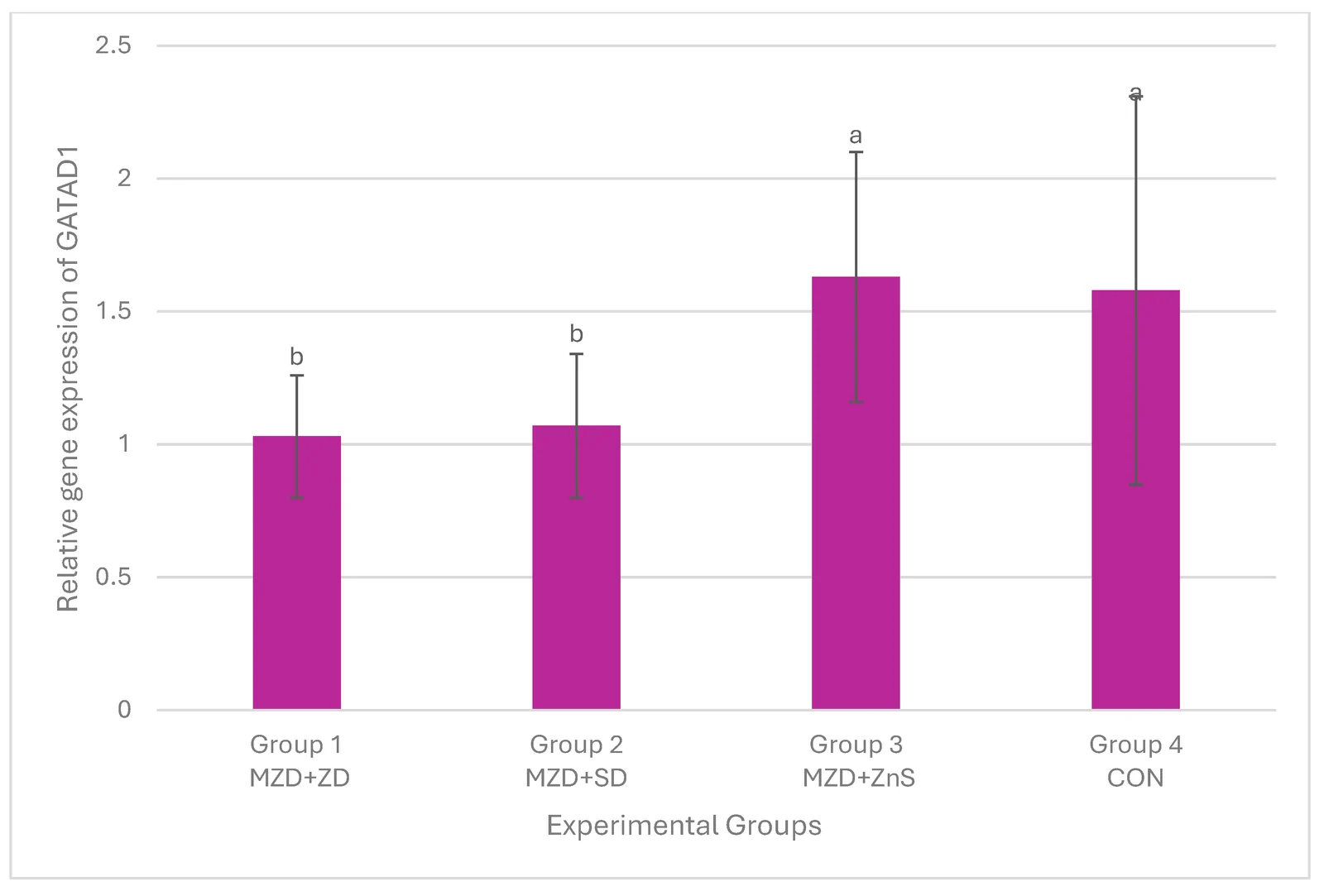
Hypothalamic GATAD1 gene expression levels of the study groups. Different lowercase letters above the data points indicate statistically significant differences among groups (*p* < 0.05). Groups marked with the same letter are not significantly different, whereas groups marked with different letters show significant differences. Specifically, in this study, the letter “a” indicates the group with the highest expression level (significantly higher than the groups marked with “b”), while the letter “b” indicates the group with a lower expression level (significantly lower than the groups marked with “a”).

**Figure 4:**
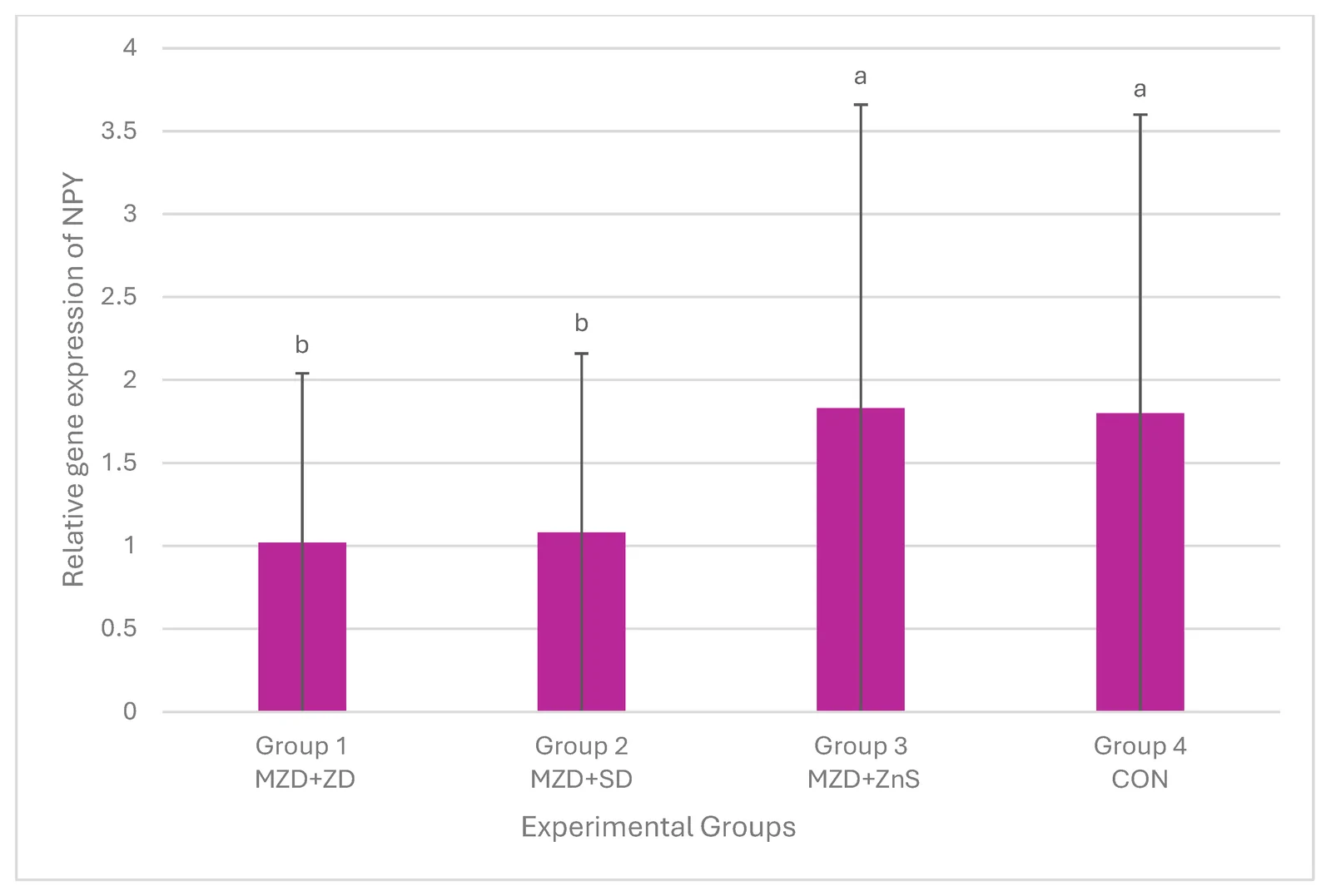
Hypothalamic NPY gene expression levels of the study groups. Different lowercase letters above the data points indicate statistically significant differences among groups (*p* < 0.05). Groups marked with the same letter are not significantly different, whereas groups marked with different letters show significant differences. Specifically, in this study, the letter “a” indicates the group with the highest expression level (significantly higher than the groups marked with “b”), while the letter “b” indicates the group with a lower expression level (significantly lower than the groups marked with “a”).

In hypothalamic gene expression analyses, we obtained the highest Kisspeptin, GATAD1, and NPY mRNA levels in G3 and G4 (*p* < 0.05). The lowest Kisspeptin, GATAD1, and NPY mRNA levels were detected in G1 and G2 (*p* < 0.05, Figure 2, Figure 3 and Figure 4 and Table 2, Table 3 and Table 4).

**Figure 5:**
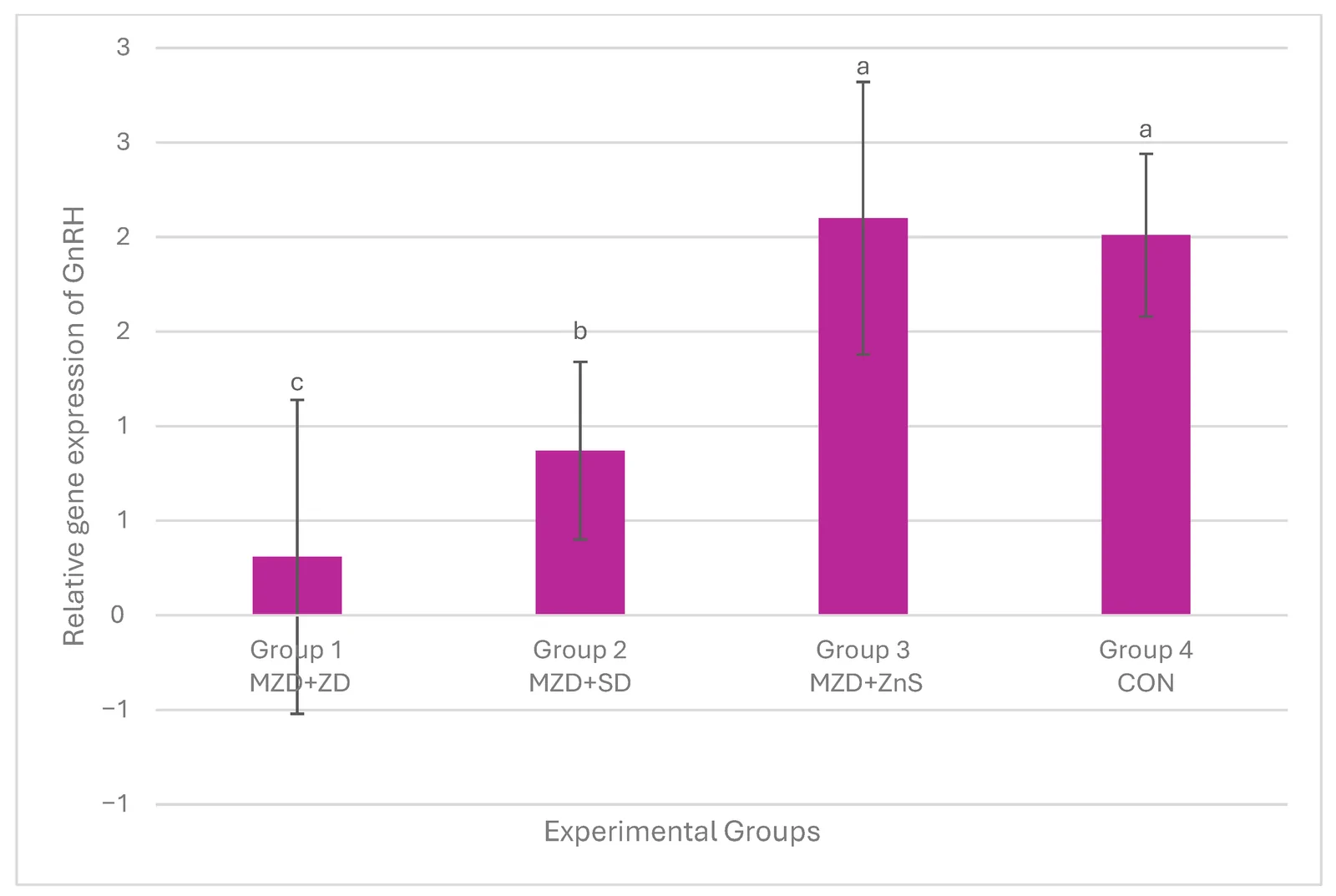
Hypothalamic GnRH gene expression levels of the study groups. Different lowercase letters above the data points indicate statistically significant differences among groups (*p* < 0.05). Groups marked with the same letter are not significantly different, whereas groups marked with different letters show significant differences. Specifically, in this study, the letter “a” indicates the group with the highest expression level (significantly higher than the groups marked with “b”), while the letter “b” indicates the group with a lower expression level (significantly lower than the groups marked with “a”).

When hypothalamic GnRH gene expression levels were evaluated, G3 and G4 had the highest mRNA levels (*p* < 0.05). The lowest GnRH gene expression was detected in G1 (*p* < 0.05). The same parameter in G2 was higher than in G1, and significantly lower than in G3 and G4 (*p* < 0.05, Figure 5 and Table 5).

### 2.3. Serum ELISA Findings

#### 2.3.1. GnRH, FSH, and LH Levels

**Figure 6:**
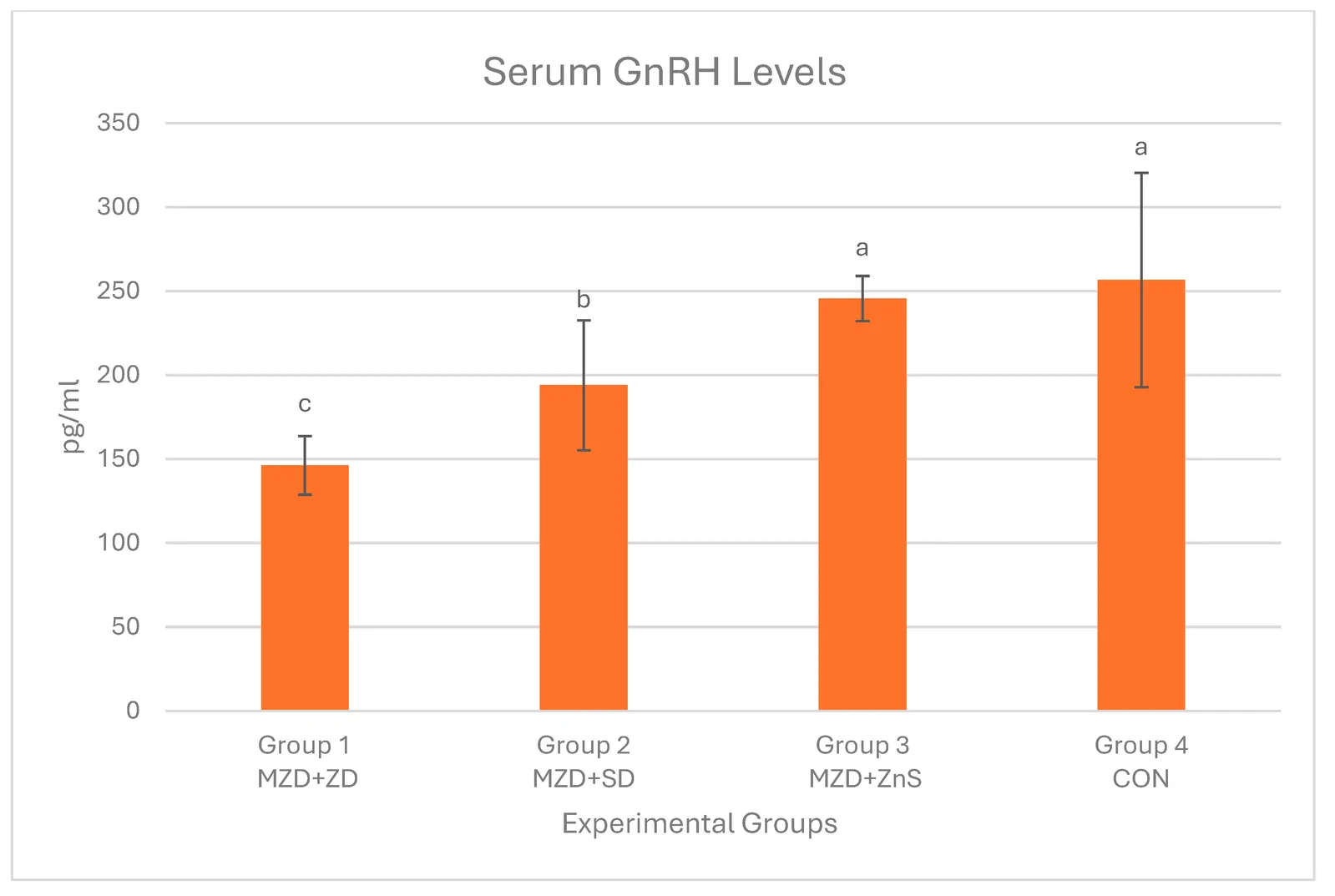
Serum GnRH levels of the study groups (pg/mL). Different lowercase letters above the data points indicate statistically significant differences among groups (*p* < 0.05). Groups marked with the same letter are not significantly different, whereas groups marked with different letters show significant differences. Specifically, in this study, the letter “a” indicates the group with the highest expression level (significantly higher than the groups marked with “b”), while the letter “b” indicates the group with a lower expression level (significantly lower than the groups marked with “a”).

**Figure 7:**
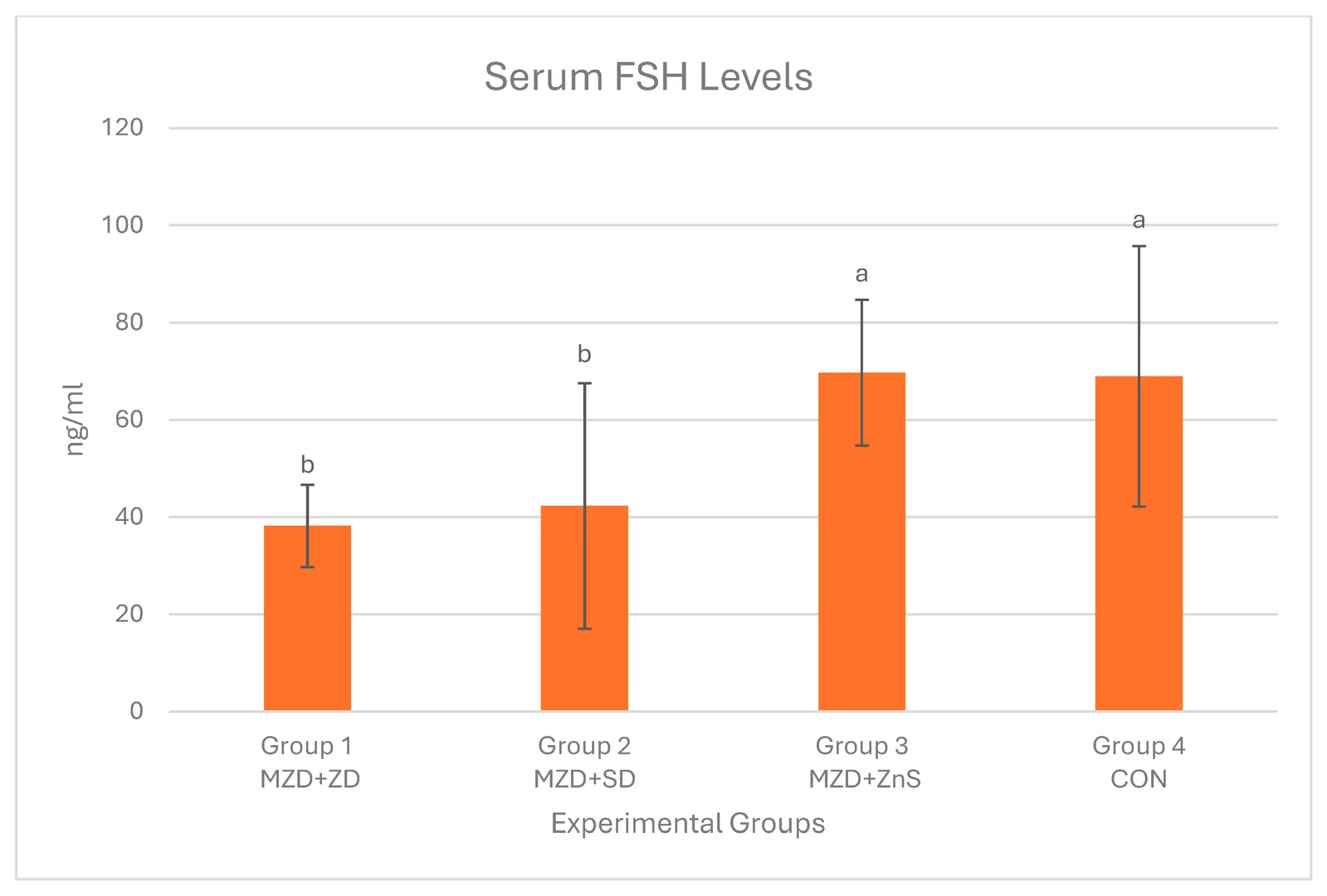
Serum FSH levels of the study groups. Different lowercase letters above the data points indicate statistically significant differences among groups (*p* < 0.05). Groups marked with the same letter are not significantly different, whereas groups marked with different letters show significant differences. Specifically, in this study, the letter “a” indicates the group with the highest expression level (significantly higher than the groups marked with “b”), while the letter “b” indicates the group with a lower expression level (significantly lower than the groups marked with “a”).

**Figure 8:**
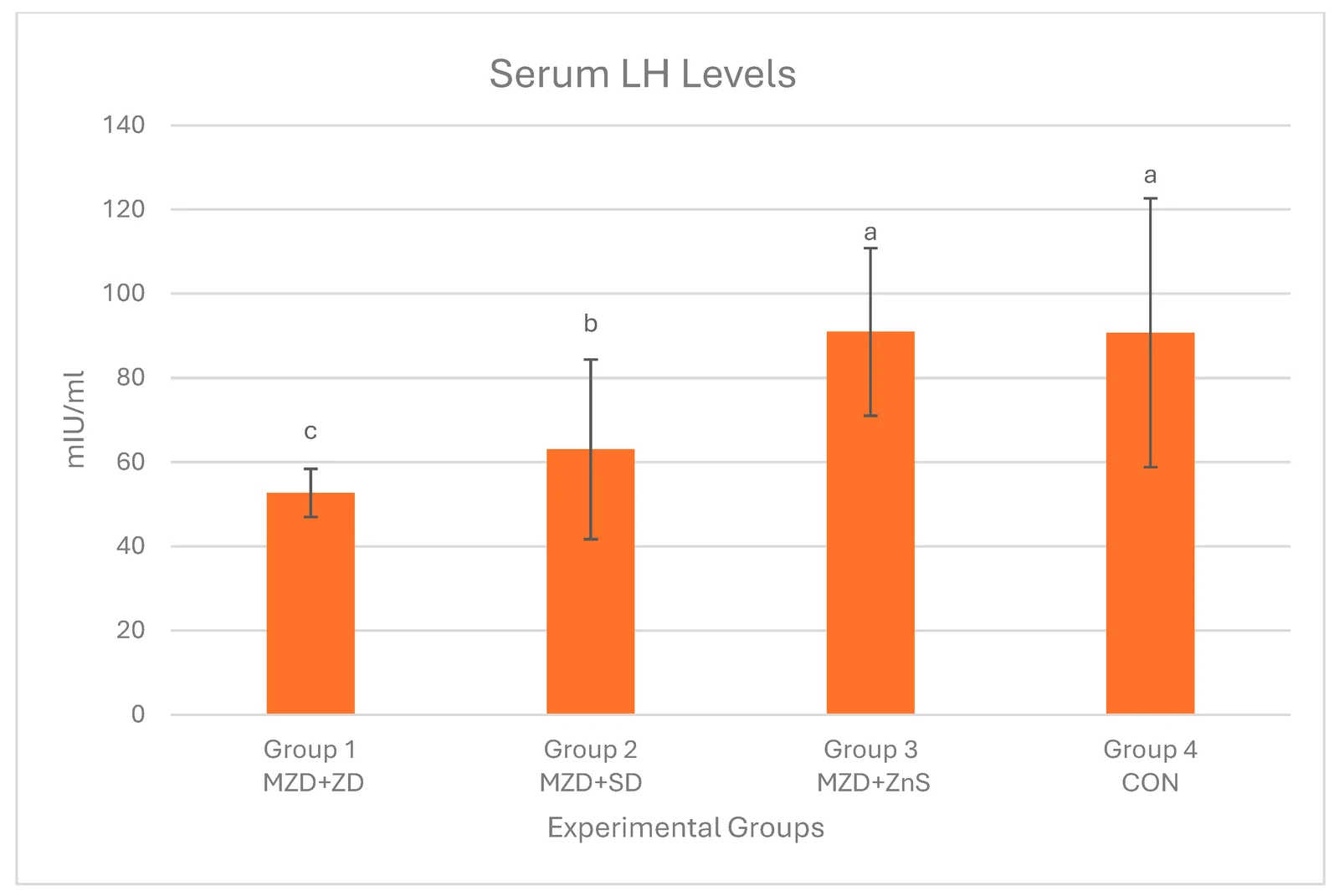
Serum LH levels of the study groups. Different lowercase letters above the data points indicate statistically significant differences among groups (*p* < 0.05). Groups marked with the same letter are not significantly different, whereas groups marked with different letters show significant differences. Specifically, in this study, the letter “a” indicates the group with the highest expression level (significantly higher than the groups marked with “b”), while the letter “b” indicates the group with a lower expression level (significantly lower than the groups marked with “a”).

Serum GnRH and FSH levels were highest in G3 and G4 (*p* < 0.05). They were lowest in G1 (*p* < 0.05, Figure 6 and Figure 7 and Table 6 and Table 7). GnRH and FSH were higher in G2 than in G1 but lower than in G3 and G4 (*p* < 0.05, Figure 3 and Figure 4). The highest serum LH levels were determined in G3 and G4 (*p* < 0.05), while the lowest LH levels were found in G1 and G2 (*p* < 0.05, Figure 8 and Table 8).

#### 2.3.2. NPY, Kisspeptin, Leptin Levels

**Figure 9:**
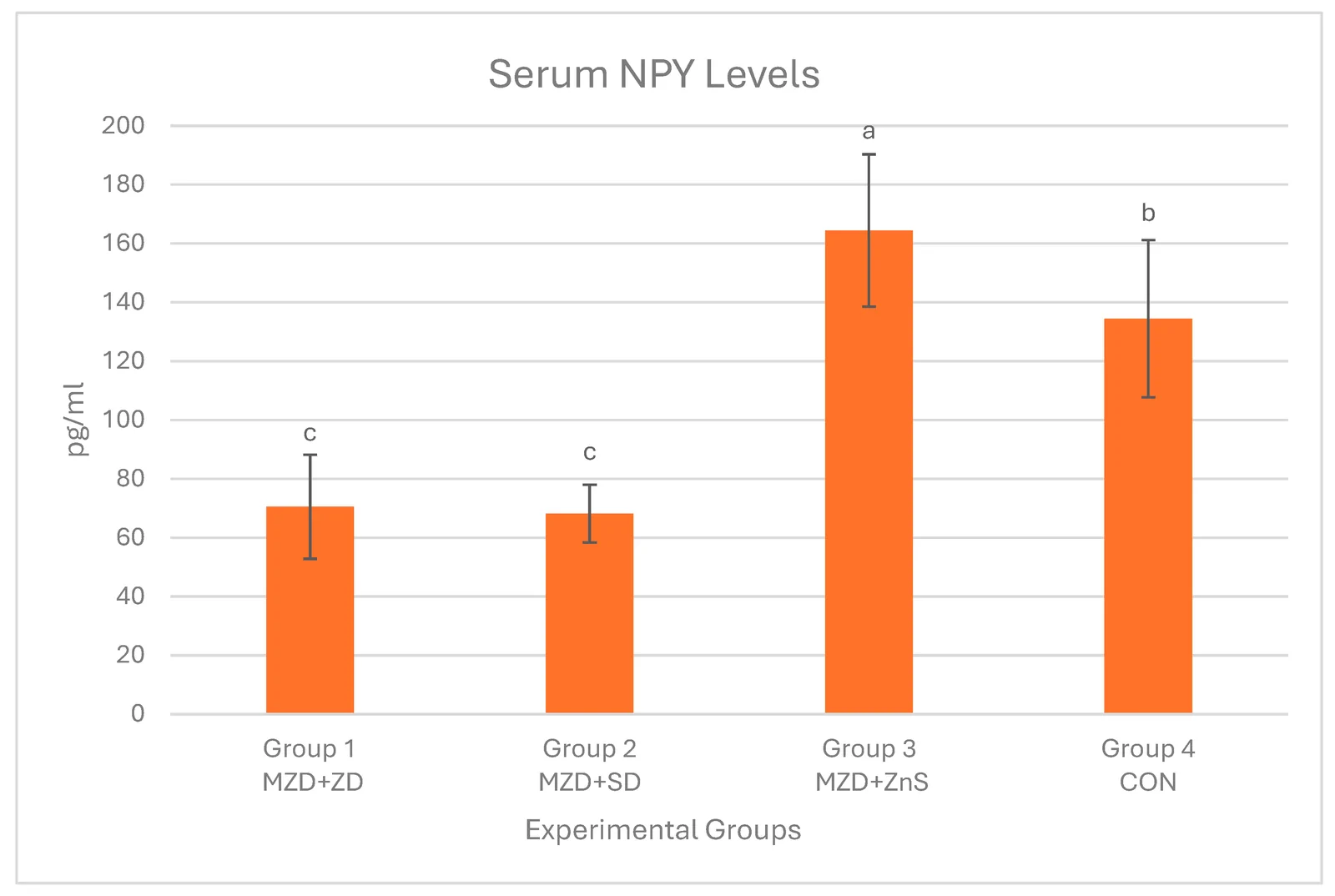
Serum NPY levels of the study groups. Different lowercase letters above the data points indicate statistically significant differences among groups (*p* < 0.05). Groups marked with the same letter are not significantly different, whereas groups marked with different letters show significant differences. Specifically, in this study, the letter “a” indicates the group with the highest expression level (significantly higher than the groups marked with “b”), while the letter “b” indicates the group with a lower expression level (significantly lower than the groups marked with “a”).

In our study, the highest serum NPY levels were detected in G3 (*p* < 0.05). Serum NPY levels in G4 were lower than in G3 (*p* < 0.05) and higher than in G1 and G2 (*p* < 0.05). Serum NPY levels in G1 and G2 were not significantly different from each other (Figure 9 and Table 9).

**Figure 10:**
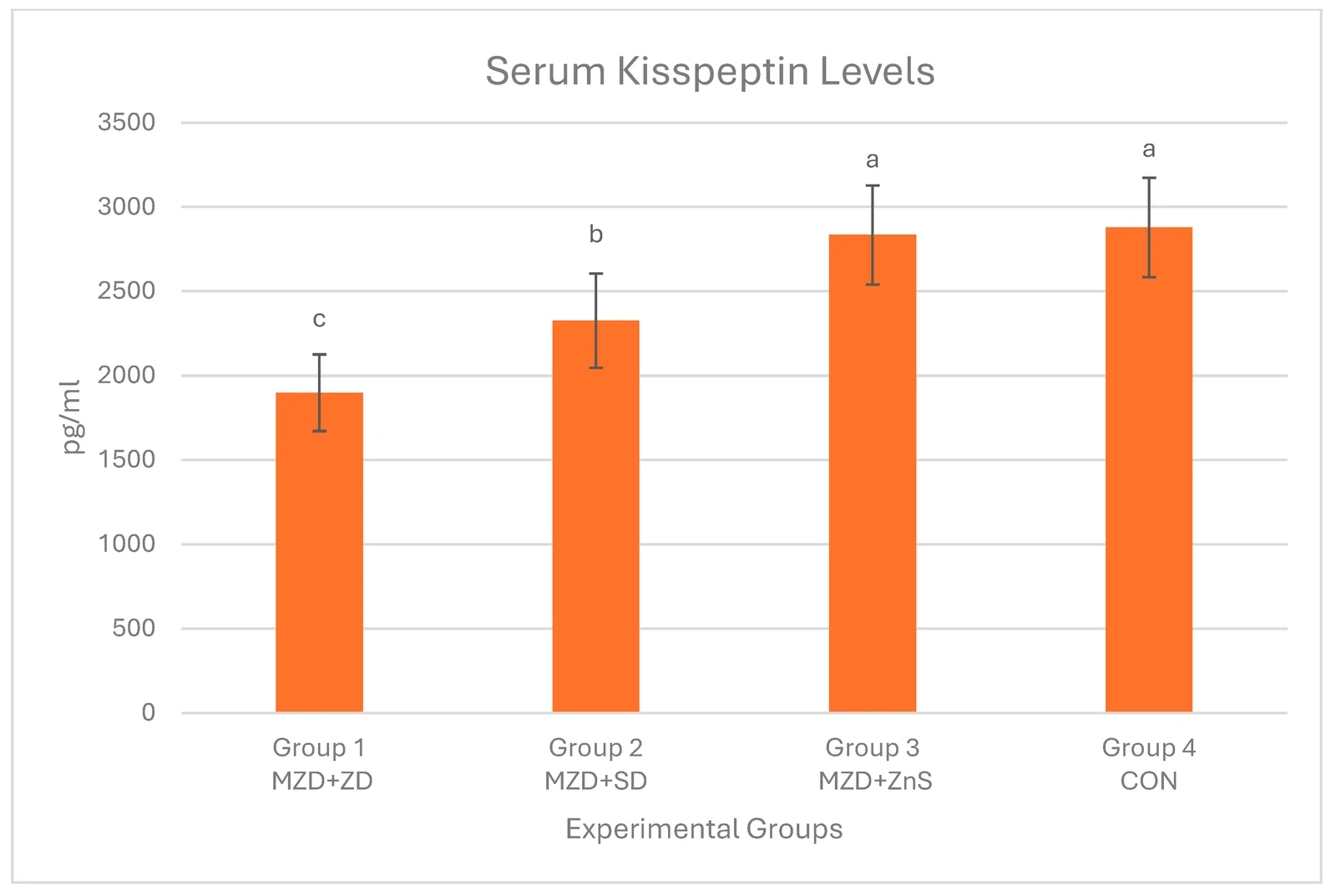
Serum kisspeptin levels of the study groups. Different lowercase letters above the data points indicate statistically significant differences among groups (*p* < 0.05). Groups marked with the same letter are not significantly different, whereas groups marked with different letters show significant differences. Specifically, in this study, the letter “a” indicates the group with the highest expression level (significantly higher than the groups marked with “b”), while the letter “b” indicates the group with a lower expression level (significantly lower than the groups marked with “a”).

In our study, the highest serum kisspeptin levels were in G3 and G4 (*p* < 0.05). The lowest serum kisspeptin level was determined in G1 (*p* < 0.05). Kisspeptin values in G2 were higher than in G1, but lower than in G3 and G4 (*p* < 0.05, Figure 10 and Table 10).

**Figure 11:**
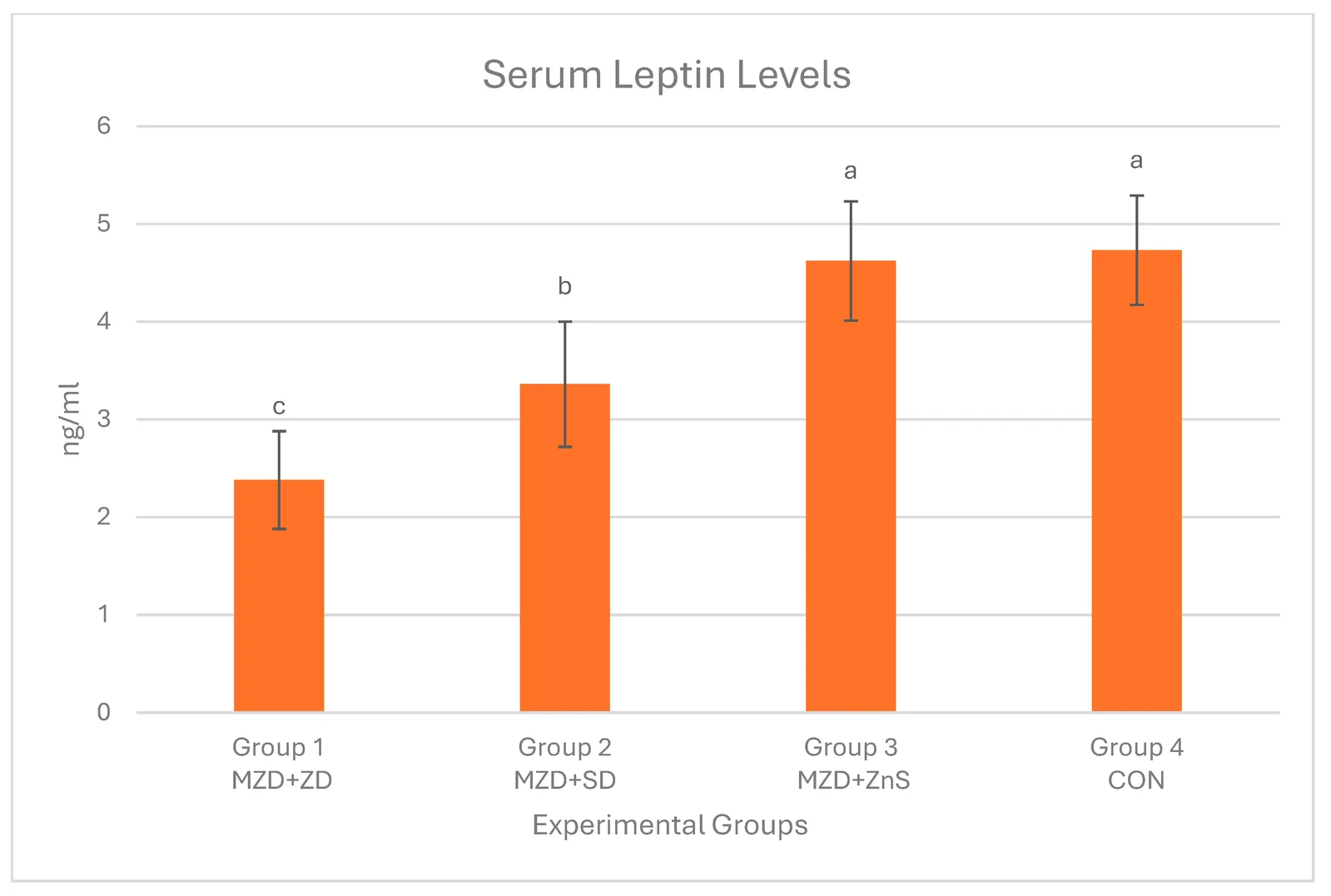
Serum leptin levels of the study groups. Different lowercase letters above the data points indicate statistically significant differences among groups (*p* < 0.05). Groups marked with the same letter are not significantly different, whereas groups marked with different letters show significant differences. Specifically, in this study, the letter “a” indicates the group with the highest expression level (significantly higher than the groups marked with “b”), while the letter “b” indicates the group with a lower expression level (significantly lower than the groups marked with “a”).

In the present study, serum leptin levels were found to be significantly lower in G1 compared to all other groups (*p* < 0.05). The highest leptin levels were found in G3 and G4 (*p* < 0.05, Figure 11 and Table 11).

## 3. Discussion

### 3.1. Evaluation of Serum Zinc Levels

In our study, the lowest serum zinc levels were detected in the G1 group (zinc-deficient diet) and the highest levels were detected in the G3 (zinc-supplemented) and G4 (control) groups, indicating that our experimental model has been successfully biochemically validated. Furthermore, the fact that serum zinc levels in the G2 group (standard diet; but born to a mother with zinc deficiency in the prenatal period) were higher than in G1 and significantly lower than in G3 and G4 suggests that maternal zinc deficiency, although partially compensated for in the postnatal period, does not completely return to normal. Zinc is an essential trace element that is tightly regulated homeostatically in the organism [19]. While serum zinc levels are sensitive to short-term dietary changes, it has been reported that deficiencies, especially in the prenatal period, can lead to permanent biochemical and molecular changes in fetal tissues [20]. This situation is associated with the limited mechanisms of zinc transport and placental transfer during the fetal period [20]. Maternal zinc deficiency has been reported to result in low serum zinc levels and impaired mineral balance in offspring, and to increase the risk of cardiovascular and metabolic diseases later in life [21]. In this respect, our zinc findings highlight the critical importance of prenatal zinc deficiency for later life. The high serum zinc levels we obtained in G3 in the present study indicate that the bioavailability of the supplementation was sufficient and that it was effectively reflected in systemic circulation. This provides an important biochemical basis for interpreting subsequent hypothalamic gene expression and serum hormone analyses. The serum zinc data of our study: confirm the effectiveness of the experimental diet model we created, show that maternal zinc deficiency can have lasting effects on the zinc levels of offspring, and furthermore, indicate that postnatal zinc supplementation can provide a systemic corrective effect.

### 3.2. Discussion of Hypothalamic GATAD1, Kisspeptin, GnRH, and NYP Gene Expression Parameters

In our study, the highest GATAD1 mRNA levels in the hypothalamus were detected in G3 (zinc supplemented) and G4 (control); the lowest were detected in G1 (zinc deficient) and G2 (prenatal deficiency + postnatal standard diet), indicating that zinc status has a significant effect on hypothalamic transcriptional regulators. GATAD1 is a transcriptional regulator with a zinc-finger structure and can play a repressive or regulatory role in gene expression through epigenetic modulation mechanisms [22]. The structural stability and DNA-binding capacity of zinc-finger proteins are largely dependent on zinc ions [22]. Therefore, zinc deficiency can disrupt not only systemic mineral balance but also the functional integrity of zinc-dependent transcription factors [23]. The significantly decreased GATAD1 expression in G1 suggests that zinc deficiency may weaken transcriptional control mechanisms at the hypothalamic level. Remarkably, GATAD1 levels remained low in G2 animals born to prenatally zinc-deficient mothers but fed a standard diet postnatally, indicating that maternal zinc deficiency could not be corrected. This suggests that epigenetic reorganization processes in the fetal period are sensitive to zinc deficiency. In contrast, the return of GATAD1 expression to control levels with zinc supplementation in G3 indicates that zinc can modulate hypothalamic gene regulatory networks not only structurally but also functionally. Considering that GATAD1 in the hypothalamus may play an indirect role in the transcriptional control of kisspeptin and GnRH neurons, which are upstream regulators of the reproductive axis [24], our results suggest that zinc status may be a critical determinant in the epigenetic regulation of the hypothalamic–pituitary–gonadal axis. Our study data regarding GATAD1 reveal that zinc deficiency suppresses zinc-dependent transcriptional mechanisms at the hypothalamic level, that maternal deficiency may not be fully compensated in the postnatal period, and that zinc supplementation can normalize GATAD1 gene expression. Because gene expression analyses were performed using whole hypothalamic tissue, the present findings do not allow identification of the specific hypothalamic nuclei contributing to the observed changes in GATAD1 expression. Therefore, the results should be interpreted as reflecting global hypothalamic expression patterns.

In our study, the highest levels of Kisspeptin mRNA in the hypothalamus were found in G3 (zinc-supplemented) and G4 (control) animals, while the lowest levels were observed in G1 (zinc-deficient) and G2 (prenatal deficiency + postnatal standard diet) groups. This indicates that zinc status directly affects the upper regulatory step of the reproductive axis. Similarly, hypothalamic GnRH mRNA levels were highest in G3 and G4, while the lowest expression was observed in the G1 group. The fact that GnRH expression was higher in the G2 group than in G1 but significantly lower than in G3 and G4 suggests that although prenatal zinc deficiency is partially compensated for in the postnatal period, complete normalization is not achieved. The hypothalamus contains two populations of kisspeptin-synthesizing neurons extending to GnRH neurons in the arcuate nucleus (ARC) and the anteroventral paraventricular nucleus (AVPV). The ARC plays a role in the negative feedback control of GnRH secretion, while the AVPV region controls positive feedback. The ARC and AVPV region kisspeptin neurons function as “GnRH pulse generators” and “GnRH surge generators,” respectively [25]. In this way, kisspeptin neurons provide the strongest excitatory input to GnRH neurons and play a decisive role in the onset of puberty [26]. A decrease in kisspeptin levels directly leads to suppression of GnRH synthesis and release. Our findings, showing a significant decrease in both kisspeptin and GnRH expression in the G1 group, indicate that zinc deficiency can suppress the hypothalamic–pituitary–gonadal axis from its initial stages [27,28]. It is also noteworthy that kisspeptin gene expression did not recover in G2 animals despite being fed a standard rat diet. This finding suggests that prenatal exposure to zinc deficiency may lead to lasting changes in the development of hypothalamic neuronal networks in the postnatal period [27]. To the best of our knowledge, the literature contains only limited data on the relationship between maternal zinc status and kisspeptin expression. A study on hybrid rams reporting that zinc supplementation led to numerical increases in kisspeptin levels [29] may partially support our findings in the current study. Although a standard postnatal rat diet slightly increased GnRH expression in G2 animals, the failure to reach control levels points to long-term neuroendocrine effects of maternal zinc status. The similarly high levels of kisspeptin and GnRH found in the zinc-supplemented G3 group suggest that zinc may be associated with neuroendocrine regulation as well as being a structural cofactor [30]. Zinc may contribute to synaptic plasticity, neurotransmitter release, and the regulation of neuroendocrine markers in the reproductive axis through zinc-dependent transcription factors. In particular, the parallel changes observed between GATAD1 expression, which was low in our previous findings, and kisspeptin and GnRH levels suggest that zinc-dependent transcriptional regulatory mechanisms may play an important role in this axis. However, the main issue in this study is that, although GATAD1 is recognized as a transcriptional regulator with potential repressive activity in certain contexts, our findings do not support a simple direct repression model for the hypothalamic Kiss1 gene in this experimental setting. Instead, GATAD1 and Kiss1 showed parallel expression patterns across the groups, suggesting coordinated regulation by zinc status and/or shared upstream epigenetic mechanisms. Therefore, in the present study, GATAD1 should be interpreted not as a definitive direct repressor of Kiss1, but rather as a zinc-dependent regulatory factor potentially involved in the broader control of adolescent neuroendocrine pathways.

Hypothalamic NPY gene expression was found to be highest in G3 and G4 and lowest in G1 and G2. This finding is evidence of a link between metabolic status and the reproductive axis in our study design. Functional in nutritional mechanisms, energy balance, and cognitive processes, NPY is also one of the main regulators of the reproductive system at the hypothalamic level [31]. The hypothalamic NPY signaling pathway is centrally important in maintaining sexual behavior and reproductive homeostasis [32]. El-bakry et al. [31] reported that zinc deficiency reduced neurotransmitter levels in the brain and also led to a significant decrease in antioxidant, monoamine, and neuropeptide Y concentrations. The decreased hypothalamic NPY gene expression we observed in zinc deficiency suggests that zinc deficiency affects not only energy balance signals but also neuronal development, limiting the functional capacity of NPY neurons [31].

Our findings related to hypothalamic gene expression highlight the critical role of zinc status in hypothalamic gene expression, energy balance, and the reproductive axis, supporting the long-term neuroendocrine importance of maternal nutrition.

### 3.3. Discussion of Serum Kisspeptin, GnRH, FSH, LH, Leptin, and NPY Levels

Our study reveals that serum measurements of Kisspeptin, GnRH, FSH, LH, Leptin, and NPY parameters demonstrate that maternal zinc deficiency and dietary zinc status lead to significant changes in offspring, particularly in leptin and NPY levels, which are involved in the kisspeptin-GnRH-gonadotropin axis and metabolic signaling.

The lowest serum kisspeptin levels in the G1 group, in parallel with hypothalamic kisspeptin gene expression findings, suggest that maternal and postnatal zinc deficiency may suppress neuronal regulatory mechanisms that play a critical role in the onset of puberty [27,28]. Kisspeptin neurons are considered one of the most potent excitatory regulators of GnRH neurons and play a fundamental role in initiating puberty [26]. Therefore, the low serum kisspeptin levels observed in the G1 group can be considered an important mechanism that may lead to suppression of the pubertal axis due to decreased GnRH secretion [26]. Indeed, the fact that GnRH levels were also found to be at their lowest in the same group in our study supports this view. In contrast, it is quite significant that kisspeptin levels were found to be high in the G3 group, where zinc supplementation was applied, similar to the control group, as was the case with hypothalamic kisspeptin gene expression in our study. To date, limited evidence has addressed the relationship between maternal zinc deficiency and/or dietary zinc status and kisspeptin. However, a report suggesting that zinc may have regulatory or supportive effects on hypothalamic neuropeptide systems [31] is consistent with the serum kisspeptin levels in our study.

In our study, parallel to the changes observed at the hypothalamic level, a similar distribution was noted in the levels of pituitary-derived gonadotropin hormones, FSH and LH. It is known that changes in GnRH secretion directly affect the release of pituitary gonadotropins. The fact that GnRH and FSH levels were highest in the G3 and G4 groups in our study indicates that adequate or increased zinc intake may support pituitary-hypothalamus-gonadal axis activity. In contrast, the significantly lower levels of both GnRH and FSH in the G1 group, where zinc deficiency was present, suggest that zinc may play an important role in the regulation of gonadotropin secretion [33]. Similarly, the lower LH levels in the zinc-deficient groups are evidence that zinc deficiency may affect different stages of the reproductive axis [33]. These results are consistent with previous studies supporting the regulatory role of zinc on neuroendocrine functions and the reproductive system [34].

The significantly lower levels of leptin, a key hormone reflecting the relationship between energy metabolism and reproductive functions, in the G1 group suggest that maternal zinc deficiency may affect puberty through metabolic signals [31]. Leptin is an adipokine that plays a critical role in the transmission of energy reserves to the hypothalamus and is considered one of the necessary metabolic signals for the onset of puberty [35]. The decrease in leptin levels caused by zinc deficiency may weaken the communication between energy balance and the reproductive axis [36]. In contrast, the fact that leptin levels were found to be high in the G3 group, where zinc supplementation was applied, similar to the control group, indicates that adequate zinc intake may play an important role in maintaining metabolic and endocrine homeostasis [36,37]. However, these findings should still be interpreted within the current understanding that leptin primarily exerts a permissive effect on pubertal development rather than acting as an independent trigger of puberty.

Another noteworthy finding is that NPY levels, another important neuropeptide evaluated in our study, were highest in the G3 group. NPY is one of the key neuropeptides that plays a role in hypothalamic energy balance and appetite regulation and may also be effective in the regulation of reproductive functions in relation to metabolic status [32,33]. The increased NPY levels in the zinc-supplemented group suggest that zinc may have modulatory effects on hypothalamic neuropeptide systems [36]. In contrast, the lower NPY levels in zinc-deficient groups suggest that zinc deficiency may suppress hypothalamic neuropeptide activity [31].

Although statistically significant differences were observed in the study, the absence of phenotypic differences does not negate the originality of the study. Although significant differences were detected in the expression levels of some target genes, no corresponding phenotypic differences were observed among the experimental groups. This finding may indicate that transcriptional alterations alone were not sufficient to produce measurable changes at the organismal level. Gene expression changes can be modulated by post-transcriptional and post-translational regulatory mechanisms, and compensatory biological processes may mitigate their functional consequences. Furthermore, complex phenotypic traits are typically controlled by multiple genes and their interactions with environmental factors. Therefore, the observed molecular responses may represent early or subtle biological adaptations that did not translate into detectable phenotypic alterations under the conditions of the present study.

## 4. Materials and Methods

### 4.1. Ethical Approval

This study was conducted at the Selçuk University Experimental Medicine Research and Application Center (SÜDAM). The research protocol was approved by the SÜDAM Animal Experiments Local Ethics Committee with decision number 2019/72. Female Wistar rats and female offspring obtained from these rats were used in the study.

### 4.2. Pregnancy Process, Grouping, and Weight Changes

Fifteen adult female rats were randomly selected to form the experimental groups, and five adult female rats were randomly selected for the control group. The mating process was carried out using the “harem method”. Male rats were removed from the cages after 10 days. Pregnancy monitoring was performed twice a day (08:00 and 17:00). The presence of a vaginal plaque was considered embryonic day 0. The 15 rats whose pregnancies were confirmed were fed a zinc-deficient diet (2.80 mg/kg Zn) from the beginning of pregnancy until the end of lactation (day 21). Female offspring were randomly selected from the available litters within each experimental group. The individual pup was considered the experimental unit for statistical analyses. Although litter effects were not modeled as a random factor, all offspring were derived from multiple dams maintained under the same experimental conditions. Female pups weaned from nursing mothers (n = 10/group) were divided into four main groups as follows:

Group 1 (MZD + ZD): Female pups born to dams fed a zinc-deficient diet during pregnancy and lactation and therefore exposed to zinc deficiency until weaning at postnatal day 21. After separation from their mothers, the pups continued to receive a zinc-deficient diet (2.80 mg/kg Zn) for an additional 45 days [38].

Group 2 (MZD + SD): Female pups born to dams fed a zinc-deficient diet during pregnancy and lactation and therefore exposed to zinc deficiency until weaning at postnatal day 21. After separation from their mothers, the pups were fed standard rat food (95.18 mg/kg, zinc) for 45 days [38].

Group 3 (MZD + ZnS): Female pups born to dams fed a zinc-deficient diet during pregnancy and lactation and therefore exposed to zinc deficiency until weaning at postnatal day 21. After separation from their mothers, the pups received a standard diet together with intraperitoneal (i.p.) zinc supplementation (5 mg/kg/day) for 45 days [38].

Group 4 (CON): Control female pups whose mothers were fed standard formula and continued to be fed standard rat food after weaning.

As expected at the start of the study, pups born to mothers fed a zinc-deficient diet had significantly lower weights than the control group. At the end of the interventions, as expected, only animals in group 1 fed a zinc-deficient diet had lower body weights than the other groups (Table 12 and Table 13). These data indicate that the dietary intervention in the study was successful.

### 4.3. Housing Conditions and Feeding Procedure

Experimental animals were housed in special steel cages sterilized daily, in a 12 h dark/12 h light cycle, and at a room temperature of 21 ± 1 °C. Standard pellet feeds were obtained from SÜDAM (Konya, Turkey), and zinc-deficient special diets were obtained from Arden Research and Experimentation (Ankara, Turkey). Fresh drinking water was provided ad libitum (freely) through glass bottles throughout the experiment.

#### Zinc Administration

Rats comprising G3 were given zinc supplementation (5 mg/kg/day) by intraperitoneal (ip) injection for 45 days.

### 4.4. Collection of Blood and Tissue Samples

Twenty-four hours after completing the applications, all animals in all groups were given a combination of intramuscular ketamine hydrochloride (Ketalar, Parke-Davis, Detroit, MI, USA) and xylazine (Rompun, Bayer, Leverkusen, Germany). and underwent general anesthesia. After the animals were sacrificed, their skulls were separated from the muscle and skin layers and then cut carefully to avoid damaging the brain tissue on both sides. The cut piece was removed with blunt forceps. The exposed brain tissue was then removed through the foramen magnum using blunt forceps. Prior to sacrifice, blood samples were collected via cardiac puncture. The collected tissue and blood samples were stored at −80 °C until analysis.

### 4.5. Real-Time PCR Analysis

#### 4.5.1. RNA Isolation

Total RNA isolation from hypothalamus tissues was performed using a commercial RNA isolation kit according to the manufacturer’s protocol. 25–50 mg tissue samples stored at −80 °C were homogenized with lysis buffer using a homogenizer (SONOPULS mini20, BANDELIN, Berlin, Germany). Phase separation was achieved with chloroform, and the supernatant obtained after centrifugation was mixed with ethanol and transferred to an RNA-binding column. Column washing steps were performed with the RPE buffer provided by the kit, and RNA was eluted with RNase-free water. The obtained total RNA samples were stored at −80 °C.

#### 4.5.2. Extraction of cDNA from mRNA

cDNA synthesis was performed by taking equal amounts from isolated total RNA samples and using the OneScript Plus cDNA Synthesis Kit (ABM, Richmond, ON, Canada) with a Thermal Cycler (Bio-Rad, Hercules, CA, USA) according to the manufacturer’s protocol. The obtained cDNA samples were stored at −80 °C until real-time PCR analysis.

#### 4.5.3. Determination of Gene Expression Levels

First, serial dilutions (1/1–1/32) were prepared from cDNA samples of the control group, and standard curves were created. Primers for the target genes and GAPDH were designed using the NCBI Primer-BLAST tool (https://www.nih.gov, accessed on 27 October 2019) based on Rattus norvegicus reference sequences and synthesized by Sentebiolab (Ankara, Turkey) (Table 14). Approximately 50 ng of each primer was used in the reactions. The FastStart Essential DNA Green Master (Roche, Basel, Switzerland) kit was preferred for amplifications, and reaction mixtures were prepared according to the manufacturer’s protocol. Real-time PCR analyses were performed on the CFX96 Touch™ Real-Time PCR Detection System (Bio-Rad, USA) using the recommended heat protocol. GAPDH was used as the endogenous reference gene for normalization. Examination of the GAPDH Cq values across the experimental groups revealed no systematic group-dependent variation, supporting its use as a reference gene under the present experimental conditions. Standard curves generated from serially diluted cDNA were used to verify linear amplification and comparable amplification performance between the target genes and GAPDH.

The obtained Cq values were analyzed using Bio-Rad CFX Manager software. Relative gene expression levels were calculated using the comparative Cq method (2^−ΔΔCq^) described by Livak and Schmittgen [38]. For each sample, ΔCq was calculated as Cq_target gene − Cq_GAPDH. The mean ΔCq value of the control group was used as the calibrator, and ΔΔCq was calculated as ΔCq_sample − mean ΔCq_control group. Relative gene expression was subsequently expressed as 2^−ΔΔCq^ fold change [39].

### 4.6. Analysis of Biochemical Parameters

#### 4.6.1. Serum Zinc Analysis

Determination of zinc concentrations was performed using an inductively coupled plasma emission spectrophotometry (ICP-AES; Varian Australia Pty LTD, Mulgrave, Australia) atomic emission device located in the Soil Department of the Faculty of Agriculture, Selçuk University, and the unit of data was μg/dL.

#### 4.6.2. Serum Analysis of NPY, Kisspeptin, Leptin, GnRH, FSH, and LH

Protein analyses were performed using ELISA kits (Elabscience, Houston, TX, USA) validated for rat samples. Protein concentrations were measured using a BMG LABTECH microplate reader (Ortenberg, Germany). The ELISA kit components included a 96-well microplate, washing buffer (20×), standard stock solution, biotinylated rat-specific antibody (separate for each protein), HRP-conjugate, TMB substrate (Solution A and B), stop solution, and standard dilution buffer. The catalog numbers of the kits used for NPY, kisspeptin, leptin, GnRH, FSH, and LH were E-EL-M0820 (96T), E-EL-R2530, E-EL-R0582, E-EL-R0451, E-EL-R0391, and E-EL-R0026, respectively. According to the manufacturer’s specifications, the assay sensitivities were 18.75 pg/mL for NPY, 46.88 pg/mL for kisspeptin, 0.10 ng/mL for leptin, 9.38 pg/mL for GnRH, 1.88 ng/mL for FSH, and 0.94 mIU/mL for LH. The intra-assay and inter-assay coefficients of variation were 4.11–6.81% and 6.36–8.26% for NPY, 5.58–7.00% and 6.29–8.57% for kisspeptin, 4.02–5.69% and 6.27–7.83% for leptin, 4.40% to 5.20% and 3.70% to 6.39% for GnRH, 4.43–5.47% and 6.79–8.91% for FSH, and 4.84–6.83% and 6.25–8.39% for LH, respectively.

### 4.7. Statistical Analysis

GraphPad Prism version 9.0.0 for Windows (GraphPadSoftware, San Diego, CA, USA) was used for statistical analyses. The parameters measured in the study were compared using one-way analysis of variance (ANOVA) and Tukey’s multiple comparison test. Values were reported as mean ± standard deviation. Differences were considered statistically significant when <0.05.

## 5. Conclusions

When all findings are evaluated collectively, the results suggest that maternal and post-weaning zinc status influence neuroendocrine pathways associated with reproductive axis regulation, as reflected by alterations in hypothalamic gene expression and serum hormone profiles. Furthermore, zinc supplementation partially or completely reversed many of these alterations, indicating its potential to mitigate the long-term neuroendocrine and metabolic consequences of maternal zinc deficiency. These findings support the concept that zinc deficiency may produce persistent effects through developmental programming mechanisms and highlight the importance of adequate zinc nutrition during early life for the proper regulation of neuroendocrine systems related to reproductive function.

Although these findings provide evidence that maternal zinc deficiency and post-weaning zinc status influence neuroendocrine markers associated with pubertal regulation in female rats, caution is warranted when extrapolating these results to humans because species-specific physiological differences may exist.

In conclusion, the present study provides additional evidence regarding the relationship between zinc status and the kisspeptin-GnRH system, an area that remains relatively limited in the current literature. Our findings indicate that maternal zinc deficiency and post-weaning zinc status are associated with changes in neuroendocrine markers involved in the regulation of the hypothalamic–pituitary–gonadal axis. However, because direct pubertal endpoints were not assessed, these findings should be interpreted as reflecting neuroendocrine correlates of pubertal regulation rather than direct measures of pubertal onset or progression. Future studies incorporating direct assessments of pubertal development, together with investigations of GATAD1-mediated epigenetic modifications and specific DNA-binding sites, will further clarify the molecular mechanisms through which zinc influences reproductive function.

### Limitations

When evaluating the findings of this study, several limitations must be considered. First, the study was conducted only on female rat pups. Therefore, it is not possible to generalize the results to male pups or directly to humans. While the experimental animal model offers significant advantages in studying the neuroendocrine effects of maternal zinc deficiency, physiological differences between species may limit the clinical implications of the results.

Secondly, the study was limited to specific genes and hormones associated with the kisspeptin/GnRH and NPY/leptin axes. Additional metabolic and neuroendocrine pathways involved in the regulation of puberty, such as the melanocortin system, which plays a role in the regulation of puberty and energy homeostasis, have not been evaluated [40]. Therefore, the molecular mechanisms explaining the effects of zinc status on the neuroendocrine system could not be fully elucidated.

Finally, only a single dose and duration of zinc supplementation were used in the study. The lack of comparison across different doses, durations, and routes of administration limits the determination of the most effective treatment strategy for correcting neuroendocrine changes due to maternal zinc deficiency. Therefore, future studies investigating different dose–response relationships, long-term effects, and possible epigenetic mechanisms would be beneficial.

Also, although offspring were randomly selected from multiple dams, statistical analyses were performed at the individual offspring level, and litter effects were not explicitly modeled. Consequently, residual intra-litter correlations cannot be entirely excluded. Future studies using litter-based analytical approaches or mixed-effects models are warranted to confirm the robustness of the present findings.

Another limitation of the study is the absence of a baseline group sacrificed at weaning (postnatal day 21) following maternal zinc deficiency exposure. Consequently, the present design does not allow complete separation of the effects of prenatal/lactational zinc deficiency from those of post-weaning zinc interventions. Future studies including a weaning-age baseline group will be valuable for distinguishing the independent and interactive contributions of prenatal and postnatal zinc status to neuroendocrine development.

Furthermore, GATAD1 was evaluated only at the mRNA level. Since zinc deficiency may affect protein abundance, structural integrity, and DNA-binding activity independently of gene transcription, the functional consequences of the observed changes in GATAD1 expression could not be directly determined. Future studies incorporating protein-level analyses (e.g., Western blotting or immunohistochemistry) and assessments of transcriptional activity will be necessary to clarify the mechanistic role of GATAD1 in zinc-dependent neuroendocrine regulation.

In addition, maternal care behaviors and lactational performance were not evaluated. Since maternal zinc deficiency may influence offspring development indirectly through alterations in maternal behavior or nursing efficiency, these factors cannot be completely excluded as contributors to the observed outcomes.

An additional limitation concerns the interpretation of circulating GnRH and NPY measurements. Because GnRH is released primarily into the hypothalamic-pituitary portal circulation and undergoes rapid degradation, serum GnRH concentrations may not directly reflect hypothalamic GnRH secretory activity. Similarly, circulating NPY levels originate from multiple peripheral and central sources and therefore may not represent hypothalamic NPY function. Accordingly, these serum measurements should be interpreted as peripheral biomarkers associated with neuroendocrine status rather than direct indices of hypothalamic peptide release.

One final limitation is that gene expression analyses were performed on whole hypothalamic homogenates. Consequently, it was not possible to determine whether the observed alterations in GATAD1, Kiss1, GnRH, or NPY expression originated from specific hypothalamic nuclei or neuronal populations. Future studies using anatomical localization techniques will be required to define the neuroanatomical specificity of these changes.

Nevertheless, this study is one of the few that evaluates the effects of maternal zinc deficiency on hypothalamic mechanisms associated with puberty and feeding regulation in offspring at both molecular and hormonal levels, and provides significant experimental evidence for zinc-kisspeptin-GnRH and zinc-NPY-leptin relationships.

## Figures and Tables

**Table 1:** Serum zinc levels of the study groups (µg/dL).

Groups	Mean ± SD
Group 1 MZD + ZD	75.81 ± 6.92 ^c^
Group 2 MZD + SD	102.04 ± 13.27 ^b^
Group 3 MZD + ZnS	124.95 ± 10.84 ^a^
Group 4 CON	123.68 ± 32.08 ^a^

Differences between groups indicated by different letters in the same column are statistically significant (*p* < 0.05), (a > b > c). *p*-Values: G1–G2: <0.0099; G1–G3: <0.0001; G1–G4: <0.0001; G2–G3: <0.0294; G2–G4: <0.0433; G3–G4: 0.9985.

**Table 2:** Hypothalamic kisspeptin gene expression levels of study groups (2^−ΔΔCt^).

Groups	Mean ± SD
Group 1 MZD + ZD	0.25 ± 0.15 ^b^
Group 2 MZD + SD	0.67 ± 0.45 ^b^
Group 3 MZD + ZnS	2.47 ± 1.21 ^a^
Group 4 CON	2.37 ± 0.67 ^a^

Differences between groups indicated by different letters in the same column are statistically significant (*p* < 0.05), (a > b). *p*-Values: G1–G2: 0.5716; G1–G3: <0.0001; G1–G4: <0.0001; G2–G3: <0.0001; G2–G4: <0.0001; G3–G4: 0.9885.

**Table 3:** Hypothalamic GATAD1 gene expression levels of the study groups (2^−ΔΔCt^).

Groups	Mean ± SD
Group 1 MZD + ZD	1.03 ± 0.23 ^b^
Group 2 MZD + SD	1.07 ± 0.27 ^b^
Group 3 MZD + ZnS	1.63 ± 0.47 ^a^
Group 4 CON	1.58 ± 0.73 ^a^

Differences between groups indicated by different letters in the same column are statistically significant (*p* < 0.05), (a > b). *p*-Values: G1–G2: 0.9967; G1–G3: <0.0306; G1–G4: <0.0545; G2–G3: <0.0501; G2–G4: <0.0864; G3–G4: 0.9947.

**Table 4:** Hypothalamic NPY gene expression levels of the study groups (2^−ΔΔCt^).

Groups	Mean ± SD
Group 1 MZD + ZD	1.02 ± 0.40 ^b^
Group 2 MZD + SD	1.08 ± 0.55 ^b^
Group 3 MZD + ZnS	1.83 ± 0.61 ^a^
Group 4 CON	1.80 ± 0.74 ^a^

Differences between groups indicated by different letters in the same column are statistically significant (*p* < 0.05), (a > b). *p*-Values: G1–G2: 0.9969; G1–G3: <0.02; G1–G4: <0.0257; G2–G3: <0.0331; G2–G4: <0.0421; G3–G4: 0.9996.

**Table 5:** Hypothalamic GnRH gene expression levels of the study groups (2^−ΔΔCt^).

Groups	Mean ± SD
Group 1 MZD + ZD	0.31 ± 0.83 ^c^
Group 2 MZD + SD	0.87 ± 0.47 ^b^
Group 3 MZD + ZnS	2.10 ± 0.72 ^a^
Group 4 CON	2.01 ± 0.43 ^a^

Differences between groups indicated by different letters in the same column are statistically significant (*p* < 0.05), (a > b > c). *p*-Values: G1–G2: 0.0612; G1–G3: <0.0001; G1–G4: <0.0001; G2–G3: <0.0001; G2–G4: <0.0001; G3–G4: 0.9735.

**Table 6:** Serum GnRH levels of the study groups (pg/mL).

Groups	Mean ± SD
Group 1 MZD + ZD	146.25 ± 17.43 ^c^
Group 2 MZD + SD	193.95 ± 38.77 ^b^
Group 3 MZD + ZnS	245.66 ± 13.48 ^a^
Group 4 CON	256.68 ± 63.85 ^a^

Differences between groups indicated by different letters in the same column are statistically significant (*p* < 0.05), (a > b > c). *p*-Values: G1–G2: <0.045; G1–G3: <0.0001; G1–G4: <0.0001; G2–G3: <0.0259; G2–G4: <0.005; G3–G4: 0.9209.

**Table 7:** Serum FSH levels of the study groups (ng/mL).

Groups	Mean ± SD
Group 1 MZD + ZD	38.16 ± 8.48 ^b^
Group 2 MZD + SD	42.28 ± 25.27 ^b^
Group 3 MZD + ZnS	69.68 ± 15.02 ^a^
Group 4 CON	68.92 ± 26.79 ^a^

Differences between groups indicated by different letters in the same column are statistically significant (*p* < 0.05), (a > b). *p*-Values: G1–G2: 0.9686; G1–G3: <0.0072; G1–G4: <0.009; G2–G3: <0.0233; G2–G4: <0.0285; G3–G4: 0.9998.

**Table 8:** Serum LH levels of the study groups (mIU/mL).

Groups	Mean ± SD
Group 1 MZD + ZD	52.69 ± 5.73 ^c^
Group 2 MZD + SD	63.05 ± 21.33 ^b^
Group 3 MZD + ZnS	90.95 ± 19.92 ^a^
Group 4 CON	90.74 ± 31.93 ^a^

Differences between groups indicated by different letters in the same column are statistically significant (*p* < 0.05), (a > b > c). *p*-Values: G1–G2: 0.7146; G1–G3: <0.0021; G1–G4: <0.0022; G2–G3: <0.0339; G2–G4: <0.0357; G3–G4: 0.9998.

**Table 9:** Serum NPY levels of the study groups (pg/mL).

Groups	Mean ± SD
Group 1 MZD + ZD	70.56 ± 17.68 ^c^
Group 2 MZD + SD	68.21 ± 9.8 ^c^
Group 3 MZD + ZnS	164.43 ± 25.86 ^a^
Group 4 CON	134.43 ± 26.75 ^b^

Differences between groups indicated by different letters in the same column are statistically significant (*p* < 0.05), (a > b > c). *p*-Values: G1–G2: 0.9952; G1–G3: <0.0001; G1–G4: <0.0001; G2–G3: <0.0001; G2–G4: <0.0001; G3–G4: <0.0224.

**Table 10:** Serum kisspeptin levels of the study groups (pg/mL).

Groups	Mean ± SD
Group 1 MZD + ZD	1898.7 ± 227.4 ^c^
Group 2 MZD + SD	2326.7 ± 279.7 ^b^
Group 3 MZD + ZnS	2834.7 ± 293.7 ^a^
Group 4 CON	2878.4 ± 294.6 ^a^

Differences between groups indicated by different letters in the same column are statistically significant (*p* < 0.05), (a > b > c). *p*-Values: G1–G2: <0.007; G1–G3: <0.0001; G1–G4: <0.0001; G2–G3: <0.0001; G2–G4: <0.0001; G3–G4: 0.9844.

**Table 11:** Serum leptin levels of the study groups (ng/mL).

Groups	Mean ± SD
Group 1 MZD + ZD	2.38 ± 0.50 ^c^
Group 2 MZD + SD	3.36 ± 0.64 ^b^
Group 3 MZD + ZnS	4.62 ± 0.61 ^a^
Group 4 CON	4.73 ± 0.56 ^a^

Differences between groups indicated by different letters in the same column are statistically significant (*p* < 0.05), (a > b > c). *p*-Values: G1–G2: <0.0029; G1–G3: <0.0001; G1–G4: <0.0001; G2–G3: <0.0001; G2–G4: <0.0001; G3–G4: 0.9755.

**Table 12:** Mean Body Weights of Female Rat Pups at the Beginning of the Experimental Procedures (Grams).

Groups	Mean ± SD
Group 1 MZD + ZD	68.2 ± 8.025 ^b^
Group 2 MZD + SD	67.00 ± 9.487 ^b^
Group 3 MZD + ZnS	67.7 ± 16.94 ^b^
Group 4 CON	92.6 ± 11.43 ^a^

Differences between groups indicated by different letters in the same column are statistically significant (*p* < 0.05), (a > b). *p*-Values: G1–G2: 0.9959; G1–G3: 0.9997; G1–G4: <0.0003; G2–G3: 0.9992; G2–G4: <0.0002; G3–G4: <0.0002.

**Table 13:** Mean Body Weights of Female Rat Pups at the End of the Experimental Period (Grams).

Groups	Mean ± SD
Group 1 MZD + ZD	132.4 ± 11.84 ^b^
Group 2 MZD + SD	199.00 ± 15.09 ^a^
Group 3 MZD + ZnS	195.6 ± 22.37 ^a^
Group 4 CON	207.6 ± 15.99 ^a^

Differences between groups indicated by different letters in the same column are statistically significant (*p* < 0.05), (a > b). *p*-Values: G1–G2: <<0.0001; G1–G3: <<0.0001; G1–G4: <0.0001; G2–G3: 0.9685; G2–G4: 0.6633; G3–G4: 0.3911.

**Table 14:** Primers used for target genes.

Gene	Primer Sequence	Function	Ampicon Size
KISSPEPTIN Forward	5′-GTCACCCATCCAGACTTCAATAA-3′	Target Gene	616
KISSPEPTIN Reverse	5′-GAAGAGCCACTTGGGTAGTT-3′	Target Gene	
GATAD1 Forward	5′CAAAGCTCCTGAGTCTGTTTCT-3′	Target Gene	122
GATAD1 Reverse	5′-CATAGTACGGCTTCCCATCTTG-3′	Target Gene	
GnRH Forward	5′-CCAAACACACAGTCAACAGAAC-3′	Target Gene	467
GnRH Reverse	5′-CTCTGTGTCTTGATGTCCCTTAG-3′	Target Gene	
NPY Forward	5′-TATCCCTGCTCGTGTGTTTG-3′	Target Gene	220
NPY Reverse	5′-TCGCAGAGCGGAGTAGTAT-3′	Target Gene	
GAPDH Forward	5′-GGGCCAAAAGGGTCATCATC-3′	Reference Gene	500
GAPDH Reverse	5′-AACCTGGTCCTCAGTGTAGC-3′	Reference Gene	

## Data Availability

The raw data supporting the results of this article will be provided by the authors upon request.
